# The Relationship Between Arterial Stiffness, Anthropometry, and Body Composition in Middle‐Aged Individuals: A Cross‐Sectional Study

**DOI:** 10.1155/tswj/9019090

**Published:** 2026-03-19

**Authors:** Thapanee Roengrit, Nawiya Huipao, Piyapong Prasertsri

**Affiliations:** ^1^ Institute of Medicine, Suranaree University of Technology, Nakhon Ratchasima, Thailand, sut.ac.th; ^2^ Division of Health and Applied Sciences, Faculty of Science, Prince of Songkla University, Songkhla, Thailand, psu.ac.th; ^3^ Faculty of Allied Health Sciences, Burapha University, Chonburi, Thailand, buu.ac.th

**Keywords:** abdominal fat, anthropometry, body composition, vascular stiffness

## Abstract

**Background:**

The association between abdominal obesity and arterial stiffness remains controversial. This study was aimed at investigating the associations between anthropometric indices, body composition, and arterial stiffness among middle‐aged Thai adults.

**Methods:**

A cross‐sectional study was conducted involving 153 participants, categorized based on the cardio‐ankle vascular index (CAVI) into low (CAVI < 9.0) and high‐CAVI (CAVI ≥ 9.0) groups. Traditional (body mass index [BMI], waist circumference [WC], and waist–hip ratio [WHR]) and novel (a body shape index [ABSI], abdominal volume index [AVI], body adiposity index [BAI], body roundness index [BRI], and conicity index [CI]) anthropometric indices were assessed. Body fat percentage (%BF), muscle mass, and visceral fat levels were measured via bioelectrical impedance analysis.

**Results:**

CAVI showed positive correlations with BMI (*r* = 0.201, *p* = 0.013), WC (*r* = 0.315, *p* < 0.001), WHR (*r* = 0.226, *p* = 0.005), ABSI (*r* = 0.172, *p* = 0.033), AVI (*r* = 0.313, *p* < 0.001), BRI (*r* = 0.249, *p* = 0.002), CI (*r* = 0.255, *p* = 0.001), and visceral fat level (*r* = 0.289, *p* < 0.001). Linear regression confirmed an association between CAVI and muscle mass (*β* = −0.559, *p* = 0.035) and visceral fat levels (*β* = 0.470, *p* = 0.010). High WC and WHR were significantly associated with arterial stiffness after adjusting for age, gender, and hypertension, whereas BMI and %BF were not.

**Conclusions:**

Abdominal obesity and visceral fat parameters are associated with high CAVI in middle‐aged individuals. A higher visceral fat level is linked to increased arterial stiffness.

## 1. Introduction

Obesity has emerged as a significant global public health concern, with projections indicating that by 2030, more than half of the world’s population may be overweight or obese [1]. Excess adipose tissue negatively affects endothelial function, promotes vascular inflammation, and contributes to arterial stiffness [2].

Arterial stiffness is closely associated with several health conditions, including hypertension (HT), kidney disease, coronary artery disease, and increased cardiovascular (CV) risk [3]. It is commonly assessed using markers such as pulse wave velocity (PWV), the ambulatory arterial stiffness index, and the cardio‐ankle vascular index (CAVI). Notably, CAVI, developed in Japan, is widely recognized for its ability to evaluate arterial stiffness independently of blood pressure (BP) [4], making it applicable across various pathological conditions.

Anthropometric measures are valuable, noninvasive tools for assessing obesity and predicting cardiovascular disease (CVD) [5]. Traditional indices, including body mass index (BMI), waist circumference (WC), hip circumference (HC), waist‐to‐hip ratio (WHR), and waist‐to‐height ratio (WHtR), are commonly used in clinical settings [6, 7]. However, these indices have limitations, as they do not differentiate between fat mass and lean body mass or account for fat distribution, which is more strongly linked to CV risk [8]. Moreover, their predictive accuracy varies among populations with different body compositions, influenced by factors such as race and sex [9].

To address these limitations, novel anthropometric indices have been developed, including a body shape index (ABSI) and body roundness index (BRI) [10]. ABSI standardizes WC relative to height and BMI, providing a refined estimate of central obesity and visceral adiposity [11]. BRI integrates WC and height to predict body fat percentage (%BF) and visceral adipose tissue volume [12]. Other indices, such as the abdominal volume index (AVI), body adiposity index (BAI) [13], and conicity index (CI), further enhance the assessment of abdominal obesity. AVI estimates overall abdominal volume using WC and HC; BAI correlates strongly with %BF [13]; and CI accounts for WC, weight, and height while excluding HC, making it a valuable marker of abdominal fat accumulation [14, 15].

Despite existing evidence, the relationship between anthropometric indices and arterial stiffness remains inconclusive. While some studies report positive associations between these indices and arterial stiffness [16], others find no significant correlation [17] or even negative correlations [18]. These discrepancies may stem from variations in study populations, differences in measurement techniques, or inconsistencies in statistical adjustments for confounding factors. Such variability underscores the need for further research, particularly in population‐specific contexts.

This study addresses this gap by systematically evaluating both traditional and novel anthropometric indices in relation to arterial stiffness, using CAVI as an independent marker. Focusing on middle‐aged Thai adults, this research examines how fat distribution patterns influence vascular health in this population. Specifically, the study is aimed (1) at comparing anthropometric indices between middle‐aged individuals with and without arterial stiffness, as defined by CAVI values; (2) at assessing the relationship between anthropometric indices and CAVI values; and (3) at evaluating the predictive capacity of these anthropometric indices for arterial stiffness. The findings may contribute to improved screening and early intervention strategies for CV risk assessment in Asian populations.

## 2. Methods

### 2.1. Study Population

This cross‐sectional study was conducted between June and August 2023, enrolling 153 Thai participants undergoing routine health checkups at Kutao’s health service in Hat Yai district, Songkhla province, Thailand. Participants were consecutively enrolled during this period. The inclusion criteria required individuals aged 35–65 years with stable vital signs for at least 7 days before participation. This age range was chosen to capture early arterial stiffness while minimizing the influence of advanced age–related diseases. The upper limit of 65 years allowed for a broader assessment of vascular changes from midlife to early aging, facilitating a more precise evaluation of preventive strategies before significant disease progression. Exclusion criteria included individuals taking medications for chronic diseases, including antihypertensives or lipid‐lowering drugs, or those whose body weight had fluctuated by more than 5 kg in the 3 months preceding enrollment. Additionally, individuals with a history of cancer, coronary artery disease, stroke, congestive heart failure, myocardial infarction, renal disease, or recent surgery (within 6 months) were excluded. Pregnant women were also ineligible. The study protocol was approved by the ethics committee of the Faculty of Medicine at Vajira Hospital (053/66 E), and all participants provided informed consent. The required sample size was determined using the two‐sample *t*‐test formula for comparing means:

n=2σ2zα∕2+zβ2Δ2.

Using the following values: *σ* ^2^ ﻿ (standard deviation) = 8.6 (estimated from previous studies [19]), *Z*_*α*/2_ (critical value from 5% significance level, two‐tailed test) = 1.96, *Z*_*β*_ (critical value for 80% power) = 0.84, and *Δ* (minimum meaningful difference between the group means) = 4.3:

N=262.7638.62 1.960.84+2/4.32=≈.

Thus, a minimum of 63 participants per group were required for the study. To account for a 30% dropout rate, the final target sample size was adjusted to 90 participants per group. However, due to participant withdrawal, incomplete data, and other unforeseen issues, the final study population consisted of 83 participants in the low‐CAVI group and 70 in the high‐CAVI group.

#### 2.1.1. Measurement of CAVI

CAVI was assessed using the Vasera VS‐1500 vascular screening system (Fukuda Denshi Co. Ltd., Tokyo, Japan). The CAVI was calculated using the formula CAVI = *a*[(2*ρ*/*Δ* *P*) × In(Ps/Pd) × PWV^2^] + *b*, where Ps represents systolic BP, Pd represents diastolic BP, PWV represents PWV from the heart to the ankle, *Δ* *P* represents Ps − Pd, *ρ* represents blood density, and constants *a* and *b* are used in the calculation. BP was measured after a 10‐min rest in a supine position, with oscillometric pressure cuffs applied systematically to both arms and ankles. The cuffs were positioned approximately 2 cm above the antecubital fossa on the arms and 2 cm above the medial malleolus on the ankles. Electrocardiogram (ECG) electrodes were attached to both wrists, and a pulse wave sensor was placed on the carotid and femoral arteries. A phonocardiography microphone was placed in the second intercostal space at the left sternal border to detect heart sounds [20]. CAVI was measured bilaterally, and the averaged values per participant were used for the analysis. Based on the mean CAVI values, participants were categorized into two groups: the low‐CAVI group (CAVI < 9.0) and the high‐CAVI group (CAVI ≥ 9.0) [20]. Participants with ankle‐brachial indices below 0.90 were excluded from the study due to the potential presence of occlusive disease, which could result in falsely low CAVI values.

#### 2.1.2. Anthropometric Measurement

Body weight and height were measured using a stadiometer while participants wore light clothing and no shoes. Weight was recorded to the nearest 0.1 kg and height to the nearest 0.1 cm. BMI was calculated by dividing weight (in kilograms) by the square of height (in meters). Obesity in Asian individuals was defined as having a BMI of 25.0 kg/m^2^ or higher. WC was measured at the midpoint between the lowest rib and the iliac crest, above the umbilicus level at the midaxillary. HC was measured at the widest part of the buttock with the tape positioned parallel to the floor, ensuring no pressure on the body surface. Measurements were recorded to the nearest 0.1 cm. The WHR was calculated by dividing WC (in centimeters) by HC (in centimeters).

Abdominal obesity was defined as having a WC of 90 cm or greater for men and 85 cm or greater for women [21] and a WHR of 0.90 or greater for men and 0.85 or greater for women [22]. The WHtR was calculated by dividing the WC (in centimeters) by the height (in centimeters). A WHtR of 0.56 or greater for men and 0.48 or greater for women is recognized as a measure of central obesity in Asian societies [23].

Other relevant anthropometric indices, including ABSI, AVI, BAI, BRI, BAI, and CI, were calculated using the following equations [11, 14, 16, 24]:

(11) ABSI=WCBMI23∕·height12∕,

(24) AVI=20.7WC2cm+×WC−HC2cm1000,

(16)

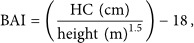

(24) BRI=364.2365.5−1−π−2×WC2m×height−2m12/,

(14) CI=WC m0.109BW kg/height m .

#### 2.1.3. Body Composition Assessments

Body composition parameters were measured using bioelectrical impedance analysis (BIA) with the TANITA UM‐076 (TANITA Corporation, Tokyo, Japan). Participants stood barefoot on the scale after removing metal accessories (e.g., belts, earrings, necklaces, and outerwear) to minimize measurement interference. The BIA method applies a high‐frequency constant current through electrodes positioned at the toes (current supply) and heels (voltage measurement) to estimate body composition. This device provided measurements for %BF, muscle mass, bone mass, and visceral fat level. The accuracy of the TANITA UM‐076 for weight is ±0.1 kg and for %BF is ±0.1%. The cutoff for %BF was set at 25% or higher for men and 35% or higher for women (commonly used in clinical practice to identify excess body fat (BF) linked to higher health risks) [24]. The visceral fat rating reflects fat stored within the abdominal cavity and is measured on a scale from 1 to 59, with higher values indicating greater accumulation. A visceral fat level exceeding 10 is considered an indicator of high CV risk factors in the Asian population [25].

## 3. Statistical Analysis

All statistical analyses were performed using SPSS. The normality of variables was assessed using the Kolmogorov–Smirnov test. Categorical variables were presented as numbers and percentages, while continuous variables with a normal distribution were presented as means and standard deviations. Skewed variables were presented as medians and interquartile ranges. The groups were compared using the chi‐squared (*χ* ^2^) test for categorical variables, the Student *t*‐test for parametric data, and the Mann–Whitney test for nonparametric data in continuous variables. Pearson correlation coefficients were used to assess simple correlations between anthropometric indices and CAVI values. Linear regression analyses were performed to examine the relationship between independent variables (baseline characteristics, anthropometrics, body composition, and BP parameters) and CAVI values. Binary logistic regression models, both unadjusted and adjusted for the covariates (age, sex, and HT), were used to assess the independent associations of the cutoff point for anthropometric indices and body compositions with the development of arterial stiffness. Odds ratios (ORs) with corresponding 95% confidence intervals (95% CIs) were computed. Statistical significance was considered at a *p* value less than 0.05.

## 4. Results

A total of 153 participants were included, with a mean age of 52 years (range: 40–64). Of the total participants, 39.87% were male and 60.13% were female. The participants were categorized into low‐CAVI (54.25%) and high‐CAVI (45.75%) groups based on their CAVI values. Additionally, 35.29% of the study population had HT. BP parameters were significantly higher in the high‐CAVI group than in the low‐CAVI group (*p* < 0.001). In contrast, the low‐CAVI group had a higher proportion of women than the high‐CAVI group (*p* = 0.048) (Table 1).

**Table 1:** Baseline characteristics of participants according to the CAVI.

**Variables**	**All (** **N** = 153**)**	**Low CAVI (** **N** = 83**)**	**High CAVI (** **N** = 70**)**	**p** **value**
Age (years)	52 (40, 64)	50 (40, 64)	53.50 (45, 62)	0.202
Female, *n* (%)	92 (60.13)	56 (67.4)	36 (51.43)	0.048 ^∗^
CAVI	8.77 ± 1.26	7.85 ± 0.69	9.85 ± 0.86	< 0.001 ^∗^
Hemodynamic parameters				
HR (beats/min)	70.00 (50.00, 100.00)	70.00 (50.00, 99.00)	70.00 (50.00, 100.00)	0.391
SBP (mmHg)	130.59 ± 16.57	124.67 ± 15.94	137.50 ± 14.60	< 0.001 ^∗^
DBP (mmHg)	83.22 ± 10.85	80.20 ± 10.50	86.80 ± 10.20	< 0.001 ^∗^
HT, *n* (%)	54 (35.29)	16 (19.28)	38 (54.28)	< 0.001 ^∗^
Anthropometry parameters				
Height (cm)	158.32 ± 8.48	157.46 ± 8.53	159.34 ± 8.35	0.171
Weight (kg)	65.00 ± 10.89	62.54 ± 9.60	67.94 ± 11.67	0.002 ^∗^
BMI (kg/m^2^)	25.53 (18.01, 47.16)	24.82 (18.01, 42.15)	25.93 (20.57, 47.16)	0.025
BMI, > 25 kg/m^2^, *n* (%)	84 (54.90)	40 (48.19)	44 (62.86)	0.075
WC (cm)	88.14 ± 9.53	85.36 ± 9.66	91.44 ± 8.29	< 0.001 ^∗^
High WC, *n* (%)	81 (52.94)	34 (40.96)	47 (67.14)	0.002 ^∗^
HC (cm)	98.00 (69.00, 131.00)	97.00 (69.00, 123.00)	99.50 (83.00, 131.00)	0.085
WHR	0.90 ± 0.07	0.88 ± 0.07	0.92 ± 0.06	< 0.001 ^∗^
High WHR, *n* (%)	95 (62.09)	40 (48.19)	55 (78.57)	< 0.001 ^∗^
WHtR	0.55 (0.42, 0.73)	0.53 (0.42, 0.73)	0.56 (0.45, 0.71)	0.001 ^∗^
High WHtR, *n* (%)	91 (59.48)	43 (51.80)	48 (68.57)	0.047 ^∗^
ABSI	0.08 ± 0.01	0.08 ± 0.01	0.08 ± 0.01	0.012 ^∗^
AVI (cm^2^)	15.24 (8.94, 26.91)	14.53 (8.94, 23.76)	16.78 (11.45, 26.91)	< 0.001 ^∗^
BAI (%)	30.88 (18.82, 53.31)	30.34 (18.82, 48.95)	31.34 (21.52, 53.31)	0.563
BRI	4.38 (1.95, 8.58)	3.89 (1.95, 8.58)	4.65 (2.43, 8.05)	0.001 ^∗^
CI	1.26 ± 0.08	1.24 ± 0.09	1.29 ± 0.07	< 0.001 ^∗^
Body composition parameters				
BF (%)	30.60 (14.00, 59.90)	29.40 (14.00, 49.50)	32.15 (14.80, 59.90)	0.426
High BF, *n* (%)	75 (49.01)	37 (44.58)	38 (54.28)	0.258
Muscle mass (kg)	39.70 (28.60, 64.60)	38.40 (28.60, 64.60)	41.60 (30.10, 64.30)	0.015 ^∗^
Bone mass (kg)	2.50 (1.60, 3.50)	2.40 (1.60, 3.50)	2.60 (1.70, 3.50)	0.009 ^∗^
Visceral fat level	9.98 ± 3.61	9.02 ± 3.24	11.11 ± 3.71	< 0.001 ^∗^
High visceral fat level, *n* (%)	74 (48.37)	32 (38.55)	42 (60.00)	0.010 ^∗^

*Note:* All variables were expressed as means ± SD, or median, and range (min, max) or number (percentage). High WC is defined as ≥ 90 cm in men, ≥ 85 cm in women; high WHR is defined as ≥ 0.90 in men, ≥ 0.85 in women; high WHtR is defined as ≥ 0.56 in men, ≥ 0.48 in women; high BF is defined as ≥ 25% in men, ≥ 35% in women; and high visceral fat level ≥ 10.

Abbreviations: ABSI, a body shape index; AVI, abdominal volume index; BAI, body adiposity index; BF, body fat; BMI, body mass index; BRI, body roundness index; CAVI, cardio‐ankle vascular index; CI, conicity index; DBP, diastolic blood pressure; HC, hip circumference; HR, heart rate; HT, hypertension; SBP, systolic blood pressure; WC, waist circumference; WHR, waist‐to‐hip ratio; WHtR, waist‐to‐height ratio.

^∗^Statistical significance was set at *p* < 0.05. *p* values were derived from independent *t*‐tests, Mann–Whitney *U* tests, or chi‐square tests, according to the characteristics of the data.

In terms of anthropometric measurements, weight, WC, WHR, WHtR, ABSI, AVI, BRI, and CI were significantly higher in the high‐CAVI group than in the low‐CAVI group, while height, HC, and BAI did not differ significantly between the two groups. Regarding BIA, muscle mass, bone mass, and visceral fat levels were significantly higher in the high‐CAVI group than in the low‐CAVI group. Within the high‐CAVI group, 67.14% had high WC, 78.57% had high WHR, 68.57% had high WHtR, 60% had a high visceral fat level, and 54.28% had HT. In addition, these parameters were significantly lower in those with normal CAVI compared to those with abnormal CAVI (*p* = 0.002, *p* < 0.001, *p* = 0.047, *p* = 0.010, and *p* < 0.001, respectively) (Table 1).

Pearson correlation analysis revealed that CAVI values were positively correlated with body weight (*r* = 0.235, *p* = 0.004), BMI (*r* = 0.201, *p* = 0.013), WC (*r* = 0.315, *p* < 0.001), HC (*r* = 0.208, *p* = 0.010), WHR (*r* = 0.226, *p* = 0.005), WHtR (*r* = 0.266, *p* = 0.001), ABSI (*r* = 0.172, *p* = 0.033), AVI (*r* = 0.313, *p* < 0.001), BRI (*r* = 0.249, *p* = 0.002), CI (*r* = 0.255, *p* = 0.001), and visceral fat level (*r* = 0.289, *p* < 0.001). However, height, BAI, %BF, muscle mass, and bone mass did not have significant correlations with CAVI values (Table 2). A multivariate linear regression analysis involving all participants revealed a significant correlation between CAVI values and muscle mass (*β* = −0.559, *p* = 0.035) and visceral fat level (*β* = 0.470, *p* = 0.010) (Table 3).

**Table 2:** The association of anthropometry indices and body composition indices with CAVI values.

**Variables**	**Correlation coefficient**	**p** **value**
Height (cm)	0.028	0.727
Weight (kg)	0.235	0.004 ^∗^
BMI (kg/m^2^)	0.201	0.013 ^∗^
WC (cm)	0.315	< 0.001 ^∗^
HC (cm)	0.208	0.010 ^∗^
WHR	0.226	0.005 ^∗^
WHtR	0.266	0.001 ^∗^
ABSI	0.172	0.033 ^∗^
AVI (cm^2^)	0.313	< 0.001 ^∗^
BAI (%)	0.117	0.150
BRI	0.249	0.002 ^∗^
CI	0.255	0.001 ^∗^
BF (%)	0.155	0.055
Muscle mass (kg)	0.052	0.521
Bone mass (kg)	0.117	0.151
Visceral fat level	0.289	< 0.001 ^∗^

Abbreviations: ABSI, a body shape index; AVI, abdominal volume index; BAI, body adiposity index; BF, body fat; BMI, body mass index; BRI, body roundness index; CI, conicity index; HC, hip circumference; WC, waist circumference; WHR, waist‐to‐hip ratio; WHtR, waist‐to‐height ratio.

^∗^Statistical significance was set at *p* < 0.05. *p* values were derived from the Pearson correlation coefficient.

**Table 3:** Multivariate linear regression showing the impact of anthropometric indices and body composition indices on CAVI.

**Variables**	**Standardized** **β**	**p** **value**
Height (cm)	−0.623	0.537
Weight (kg)	0.156	0.935
BMI (kg/m^2^)	0.352	0.831
WC (cm)	−0.351	0.908
HC (cm)	0.016	0.991
WHR	−0.086	0.932
WHtR	1.432	0.452
ABSI	0.115	0.944
AVI (cm^2^)	1.652	0.391
BAI (%)	−0.283	0.855
BRI	−3.218	0.087
CI	0.359	0.876
BF (%)	0.049	0.845
Muscle mass (kg)	−0.559	0.035 ^∗^
Bone mass (kg)	−0.012	0.972
Visceral fat level	0.470	0.010 ^∗^

Abbreviations: ABSI, a body shape index; AVI, abdominal volume index; BAI, body adiposity index; BF, body fat; BMI, body mass index; BRI, body roundness index; CI, conicity index; HC, hip circumference; WC, waist circumference; WHR, waist‐to‐hip ratio; WHtR, waist‐to‐height ratio.

^∗^Statistical significance was set at *p* < 0.05. *p* values were derived from the linear regression model.

After adjusting for potential confounders, the development of arterial stiffness was significantly associated with high WC (OR: 2.590, 95% CI: 1.227–5.464, *p* = 0.012), high WHR (OR: 2.570, 95% CI: 1.162–5.687, *p* = 0.020), and high WHtR (OR: 6.156, 95% CI: 1.877–20.195, *p* = 0.003). A high visceral fat level was significantly correlated with CAVI > 9 in the unadjusted model, but after adjusting for confounding factors, this factor was not significantly associated with arterial stiffness. However, high BMI and high BF were not associated with high CAVI (Table 4).

**Table 4:** Binary logistic regression model to determine the association between the anthropometric indices and body composition indices and the development of arterial stiffness (CAVI ≥ 9).

**Variables**	**Model 1**		**Model 2**	
	**OR (95% CI)**	**p** **value**	**OR (95% CI)**	**p** **value**
BMI, ≥ 25 kg/m^2^	1.819 (0.951–3.479)	0.070	1.456 (0.704–3.012)	0.311
WC, men ≥ 90 cm, women ≥ 85 cm	2.945 (1.517–5.718)	0.001 ^∗^	2.590 (1.227–5.464)	0.012 ^∗^
WHR, men ≥ 0.90, women ≥ 0.85	3.942 (1.928–8.058)	< 0.001 ^∗^	2.570 (1.162–5.687)	0.020 ^∗^
WHtR, men ≥ 0.56, women ≥ 0.48	2.030 (1.046–3.940)	0.036 ^∗^	6.156 (1.877–20.195)	0.003 ^∗^
BF, men ≥ 25*%*, women ≥ 35*%*	1.476 (0.779–2.797)	0.232	1.305 (0.636–2.675)	0.468
Visceral fat level ≥ 10	2.391 (1.247–4.585)	0.009 ^∗^	2.069 (0.857–4.997)	0.106

Abbreviations: BF, body fat; BMI, body mass index; CI, confidence interval; HT, hypertension; OR, odds ratio; WC, waist circumference; WHR, waist‐to‐hip ratio; WHtR, waist‐to‐height ratio.

^∗^Statistical significance was set at *p* < 0.05. *p* values were derived from the logistic regression model (Model 1: unadjusted and Model 2: adjusted for age, sex, and HT).

## 5. Discussion

In the present study, significant associations were shown between arterial stiffness, as measured by CAVI, and various anthropometric and body composition indices, particularly in visceral fat, WC, WHR, and WHtR. These findings highlight the critical role of visceral fat as a predictor of arterial stiffness, particularly in abdominal obesity, which is a significant risk factor for CV health. Moreover, abdominal obesity, characterized by high WC, WHR, and WHtR, was strongly associated with arterial stiffness, even after adjusting for confounding factors such as age, gender, and HT.

Traditional anthropometric indices are commonly used to assess the correlation between arterial stiffness and obesity‐related metabolic disorders. However, these indices have limitations in differentiating various aspects of body composition [26]. While BMI provides a general evaluation of overall BF, WC and WHR are more effective for assessing abdominal or central obesity and its associated health risks [5]. Studies have shown that WC and WHR exhibit a stronger correlation with arterial stiffness than BMI, as evidenced by their association with carotid‐femoral PWV and brachial‐ankle PWV. A retrospective study demonstrated a significant association with WHR and brachial‐ankle PWV, whereas BMI showed no such correlation [27]. Additionally, WC exhibited stronger associations with both measures than BMI [28]. In a study of healthy Japanese participants, BMI was inversely related to CAVI [29], whereas central obesity, measured by WHR, had a positive association [18]. Furthermore, among individuals with overweight, WC and WHR were positively correlated with PWV but not with endothelial function [30]. These findings align with our results, suggesting that the abdominal obesity markers, such as WC and WHR, may be more reliable indicators of arterial stiffness and CV risk than BMI alone.

WC and WHR have been independently associated with PWV and central augmentation index (cAIx) [31]. Importantly, these measures are considered superior to BMI, showing stronger associations with CVD and metabolic syndrome (MetS) [32]. While WC primarily reflects subcutaneous rather than visceral fat, some individuals with normal BMI and WC may still have excessive visceral fat, a key risk factor for metabolic disorders [33]. However, WC and WHR cannot distinguish between abdominal BF mass and visceral fat. Additionally, since these indices do not account for height, they may underestimate risk in shorter individuals and overestimate it in taller individuals [31].

To overcome the limitations of traditional anthropometric indices, particularly variations due to height, the WHtR was developed. This index reduces variability and provides a more precise assessment, especially in children and adolescents [10]. A previous study has shown a positive correlation between PWV and WHtR, supporting its reliability as a marker for arterial stiffness [30]. Furthermore, WHtR has proven to be a stronger predictor of MetS, making it a valuable tool for assessing CV risk [12].

Our findings highlight a significant association between arterial stiffness and novel anthropometric indices, such as ABSI, AVI, BRI, and CI. These indices were notably elevated in the high‐CAVI group compared to the low‐CAVI group. A retrospective cross‐sectional study demonstrated a positive correlation between CAVI and ABSI in middle‐aged, nonobese Japanese participants [34]. Additionally, an ABSI has been significantly associated with carotid intima‐media thickness, reinforcing its relevance as a CV risk marker [8]. In individuals with Type 2 DM and those with overweight or obesity, ABSI and BRI have been associated with visceral obesity and BF, respectively, making them valuable indicators of disease risk and mortality [10]. Among hypertensive individuals, a BRI greater than 4.62 has been linked to an increased risk of diabetes, suggesting its role as an independent predictor of diabetes onset [35].

Both ABSI and BRI correlate with vascular stiffness and have demonstrated superior predictive power for health risks compared to traditional measures [17]. BRI is strongly linked to visceral obesity, while ABSI exhibits a weaker correlation with arterial stiffness but improves in predictive accuracy when combined with BMI [36]. Notably, both indices are more effective than WC and BMI in predicting the risks of diabetes, HT, and CVD, further emphasizing their clinical utility in assessing mortality risk [8].

However, findings on alternative indices such as AVI, BAI, and CI remain inconsistent [15, 37]. While some studies have proposed these indices as useful markers, their associations with arterial stiffness are debated. For instance, although AVI and BRI have been positively correlated with carotid‐femoral pulse wave velocity (cf‐PWV), this relationship was not significant after adjusting for age and gender. Similarly, BAI has shown no significant link to arterial stiffness [16]. On the other hand, CI has been associated with insulin resistance and dyslipidemia—both key risk factors for arterial stiffness and HT [38]. These discrepancies may stem from variations in participant characteristics, study sample sizes, and the presence of underlying conditions, highlighting the need for further research to clarify their clinical relevance.

CAVI values were strongly associated with visceral fat, confirming its role as a reliable predictor of arterial stiffness. Visceral fat is a stronger predictor of CV risk than subcutaneous fat, promoting insulin resistance, endothelial dysfunction, and inflammation, all of which accelerate arterial stiffness^5.28^. This relationship is particularly relevant in populations with a higher prevalence of visceral fat, such as those in Asia [32].

The present study found a significant inverse correlation between muscle mass and CAVI, supporting the association between low muscle mass and increased arterial stiffness. This aligns with previous research showing reduced muscle mass is linked to higher CV risk [19], especially in long‐lived individuals. Increased arterial stiffness impairs nutrient and oxygen supply to muscles, promoting muscle loss [39]. Similar findings have been observed in Korean populations and a European study, where CAVI showed strong correlations with skeletal muscle mass, particularly in women [40, 41]. Various mechanisms, including reduced blood flow, maladaptive muscle remodeling, inflammation, insulin resistance, and oxidative stress, may explain this relationship, further exacerbating both muscle loss and arterial stiffness [42].

Although the present study did not find a direct correlation between BF and CAVI values, previous research has shown that lower BF is associated with reduced arterial stiffness in women aged 16–58 years [43]. In older adults, particularly those aged 80 years, BF has been found to directly correlate with increased arterial stiffness, contributing to higher CV risk in long‐lived individuals [44]. Additionally, age‐related associations with arterial stiffness, as indicated by aortic PWV and augmentation index (AIx%), have been observed, with older women showing higher arterial stiffness compared to younger and athletic women [43]. These findings underscore the influence of biological sex on arterial stiffness parameters.

The strength of this study lies in its comprehensive analysis of the relationship between vascular stiffness and multiple anthropometric parameters, particularly novel indices. This approach offers valuable insights into predicting arterial stiffness in middle‐aged individuals, which could support early prevention and intervention strategies to improve quality of life in later years.

However, this study has several limitations. First, it did not account for variables such as medication use, blood glucose levels, lipid profiles, or lifestyle factors (e.g., physical activity, diet, smoking, and alcohol consumption), all of which may influence the observed associations. Incorporating these variables into future analyses could strengthen the findings. Second, the cross‐sectional design prevents the assessment of temporal changes in vascular abnormalities, limiting the ability to establish causal relationships. Longitudinal studies are needed to address this gap. Third, as the study focused exclusively on Thai adults, the generalizability of the results to other ethnic groups remains uncertain. Expanding the study population could provide broader insights. Lastly, the absence of gender‐ and age‐specific analyses limits the understanding of potential demographic differences. While this study did not include such subgroup analyses, future research should explore these variations, and we plan to incorporate them in subsequent studies to enhance the comprehensiveness of findings.

In conclusion, this study found a significant association between central obesity (WC, WHR, and WHtR) and arterial stiffness in Thai participants, highlighting the role of visceral fat in CV risk. Monitoring central obesity should be a key part of CV risk assessment, especially in middle‐aged individuals. Given the rising prevalence of obesity, future research should focus on longitudinal studies to explore the causal link between visceral fat and arterial stiffness and intervention studies aimed at reducing abdominal fat to improve vascular health.

## Conflicts of Interest

The authors declare no conflicts of interest.

## Author Contributions

Conceptualization: Thapanee Roengrit. Data curation: Thapanee Roengrit and Nawiya Huipao. Methodology: Thapanee Roengrit, Nawiya Huipao, and Piyapong Prasertsri. Writing–original draft: Thapanee Roengrit. Writing–review and editing: Thapanee Roengrit, Nawiya Huipao, and Piyapong Prasertsri.

## Funding

No funding was received for this manuscript.

## Data Availability

The data that support the findings of this study are available from the corresponding author upon reasonable request.
